# Influences on Attitudes Regarding Potential COVID-19 Vaccination in the United States

**DOI:** 10.3390/vaccines8040582

**Published:** 2020-10-03

**Authors:** Kendall Pogue, Jamie L. Jensen, Carter K. Stancil, Daniel G. Ferguson, Savannah J. Hughes, Emily J. Mello, Ryan Burgess, Bradford K. Berges, Abraham Quaye, Brian D. Poole

**Affiliations:** 1 Department of Microbiology and Molecular Biology, Brigham Young University, Provo, UT 84602, USA; kendallpogue@gmail.com (K.P.); carterstancil@gmail.com (C.K.S.); savannahhughes003@gmail.com (S.J.H.); emilyjmello@gmail.com (E.J.M.); ryanburgess1517@gmail.com (R.B.); brad.berges@gmail.com (B.K.B.); quayeabraham29@gmail.com (A.Q.); 2 Department of Biology, Brigham Young University, Provo, UT 84602, USA; Jamie.Jensen@byu.edu (J.L.J.); danferg21@gmail.com (D.G.F.)

**Keywords:** COVID-19, vaccine hesitancy, vaccine attitudes, vaccine development, SARS-CoV-2

## Abstract

The COVID-19 pandemic continues to ravage the world, with the United States being highly affected. A vaccine provides the best hope for a permanent solution to controlling the pandemic. However, to be effective, a vaccine must be accepted and used by a large majority of the population. The aim of this study was to understand the attitudes towards and obstacles facing vaccination with a potential COVID-19 vaccine. To measure these attitudes a survey was administered to 316 respondents across the United States by a survey corporation. Structural equation modeling was used to analyze the relationships of several factors with attitudes toward potential COVID-19 vaccination. Prior vaccine usage and attitudes predicted attitudes towards COVID-19 vaccination. Assessment of the severity of COVID-19 for the United States was also predictive. Approximately 68% of all respondents were supportive of being vaccinated for COVID-19, but side effects, efficacy and length of testing remained concerns. Longer testing, increased efficacy and development in the United States were significantly associated with increased vaccine acceptance. Messages promoting COVID-19 vaccination should seek to alleviate the concerns of those who are already vaccine-hesitant. Messaging directed at the benefits of vaccination for the United States as a country would address the second predictive factor. Enough time should be taken to allay concerns about both short- and long-term side effects before a vaccine is released.

## 1. Introduction

The COVID-19 pandemic has inflicted almost unimaginable harm on the life, health and economy of many nations. Along with hygienic and behavioral control measures, vaccination is the most successful way of limiting or eliminating viral infection and spread. Although the exact timing of when a vaccine against COVID-19 will be available is unknown, several candidates are being pursued, and it is likely that at least one effective vaccine will soon become available [1]. States in the United States are being told by the Centers for Disease Control and Prevention to prepare for a vaccine by November 1 [2], which would likely not allow adequate time for FDA approval.

Even the best vaccine cannot be effective if it is not used. Recent surveys found that only 49% and 70% of residents of the United States of America plan to receive a COVID-19 vaccine when available [3,4]. This number of participants is likely below the threshold needed for homogeneous herd immunity [5], and will leave many residents vulnerable to the disease, even with a vaccine available. The problem of vaccine refusal has many contributing factors and is present worldwide [6,7]. The World Health Organization (WHO) recommends a preemptive strategy to overcome vaccine hesitancy and build trust in a vaccine to prepare for maximum efficacy when a vaccine is available [8,9]. Controlling or ending the pandemic through a vaccination program requires an understanding of the reasons behind hesitancy towards a COVID-19 vaccine in the United States, as well as strategies to overcome this hesitancy. Understanding varying vaccine attitudes is especially important since a heterogeneous approach to vaccine refusal that deals with the concerns of different groups is more effective than a homogenous strategy [10,11,12]. A qualitative and quantitative understanding of attitudes towards vaccine development and confidence is especially urgent in the United States, given recent messages that COVID-19 vaccine development is being accelerated and a vaccine may be released even before clinical trials are completed.

Several strategies exist for ameliorating vaccine hesitancy. Our previous work explored strategies for improving opinions about vaccines among vaccine-hesitant college students. We found that focusing on the physical, social and emotional impacts of the diseases, either by having students interview someone who had suffered from a vaccine-preventable disease, or by taking a course with a heavy focus on the diseases that can be prevented by vaccination, significantly improved attitudes towards vaccines in the vaccine-hesitant students [13]. A study of elderly adults in the United States found that access to health information was a positive predictor for receiving vaccines [14]. In a very large study of United States adults, hesitancy towards the influenza vaccine was found to be much higher than hesitancy towards vaccines in general, an attitude resulting primarily from concerns about efficacy [15]. The low level of confidence in a COVID-19 vaccine is likely a result of multiple factors found to influence confidence regarding other vaccines, such as confidence in efficacy of the vaccine, fear of side effects [16], and trust in the government and those developing and administering the vaccines [17]. Lower economic status and lower education have also been associated with vaccine refusal [18]. Since people are currently experiencing the COVID-19 pandemic in real-time, there is unfortunately an opportunity to investigate the role of personal experience with disease, demographic factors, social conscience and the development, timing and nature of the vaccine itself in vaccine hesitancy.

We designed a survey using structural equation modeling to investigate multiple potential contributing factors to COVID-19 vaccine refusal. The survey was distributed throughout the United States. We hypothesized that personal experience with COVID-19, whether being diagnosed personally or knowing someone who was diagnosed, would be important in determining the participants’ attitudes towards the vaccine. Considerations such as the closeness of the relationship to a diagnosed person and the severity of their disease were considered as part of this hypothesis. We also hypothesized that an individual’s knowledge about the SARS-CoV-2 coronavirus and COVID-19 disease would also be a determining factor towards COVID-19 vaccine attitudes. Furthermore, we expected that an individual’s attitudes towards vaccines in general would be an important factor in their willingness to receive a COVID-19 vaccine when one becomes available. Overall vaccine hesitancy is likely to play a large role in COVID-19 vaccine hesitancy, especially since vaccine hesitancy has been growing in more than 90% of countries [19] and has now been identified by the WHO as one of the greatest threats to global health. The model also included demographic factors and attitudes towards the severity of the COVID-19 pandemic. Along with the modeled predictors of vaccine attitude, we examined how the timing of the vaccine development process, the effectiveness of the final vaccine, and the location where the vaccine is developed would affect attitudes towards a COVID-19 vaccine.

## 2. Materials and Methods

### 2.1. Measures

We constructed and administered a survey to measure multiple factors related to the impact of COVID-19, the opinions and knowledge of participants about the disease and about vaccines and the intention and behavior of participants regarding a potential COVID-19 vaccine and vaccines in general, as well as demographic factors such as age, race, sex, and indicators of income. Additional items were included to evaluate factors such as the length of testing respondents perceived to be effective, how efficacy affects the likelihood to vaccinate, and how the location of vaccine development affects comfort with the vaccine.

The survey was administered by Qualtrics (Provo, UT, USA) across the United States. In total 316 responses were collected according to census demographics (see demographics section for further details). The survey was administered via email notification through the Qualtrics survey panel using an anonymous link, and once the 316 responses were collected, the survey was closed.

Some of these items were used to measure specific latent variables hypothesized to have a relationship with a person’s intent to get a COVID-19 vaccine, should it become available. These latent variables are as follows: (1) history of vaccination, (2), underlying knowledge of vaccine immunity, and (3) attitudes and intentions toward a COVID-19 vaccine. Further details of each latent variable will be described in the Results section. Covariates measuring the personal impact of COVID-19, demographics, and perceived impacts of the pandemic on America were included in the model.

Participants were selected by age, race and sex to reflect national census data. Only completed surveys were used for analysis. Quality control was performed using a timing method, whereby any participant who spent less than half the mean time completing the survey was rejected. The project was approved by the BYU Institutional Review Board (IRB# IORG0001302, Protocol # RB2020-342). The survey is available in the Supplementary Materials.

### 2.2. Sample and Demographics

The survey garnered 316 responses that were selected to reflect national census data. Demographic information is presented in Table 1. Responses were collected from across the United States.

### 2.3. Statistical Analysis

#### 2.3.1. Model Analysis

First, we used factor analysis and Structural Equation Modeling (SEM) to determine the relationships between multiple variables. Mplus software ver. 8 (Muthen and Muthen, 1998–2010, Los Angeles, CA, USA) was used to perform both confirmatory factor analysis (CFA) on the measurement portion of our model and SEM on the structural portion of our model. For each latent variable, a CFA was performed with a request for modification indices. Then items were removed until fit indices (root mean square error of approximation (RMSEA), comparative fit index (CFI), Tucker-Lewis index (TLI), standardized root mean square residual (SRMR), and Chi square (χ^2^)) were acceptable. Then all instruments were combined into a full measurement model to ensure model fit prior to structural modeling. Following combination into the model, our hypothesized structural model was used, and SEM was performed, including several other items from the survey as covariates. These covariates are as follows: the respondents’ perception of the impact of COVID-19 on the United States of America (perceived impact on America) (respondents were asked to rate the statement, “How much of a problem is COVID-19 in America?” on a 5-point Likert scale from “Not a problem at all” to “The most important problem facing America right now”), the number of exposures to COVID-19 (we asked respondents to indicate how many individuals they knew who had had COVID-19), household income (respondents were asked their total household income before taxes and were binned into intervals) and political ideology (respondents were asked to select from the following categories: very conservative, somewhat conservative, neither conservative nor liberal, somewhat liberal, very liberal). We used the full information maximum likelihood method to deal with missing data. The final model was selected based on fit statistics, as described in the Results.

#### 2.3.2. Other Analysis

For questions not involved in the structural equation model, repeated-measures ANOVA was used to test for variance between multiple conditions. The Wilcoxon Signed rank test was used to detect significance between groups. Pearson’s R test was used to evaluate correlations.

## 3. Results

### 3.1. Statistical Results of CFA and SEM

#### 3.1.1. Confirmatory Factor Analysis (CFA)

We used CFA to confirm that each latent factor was being measured appropriately by each item. Items were removed until appropriate fit statistics were obtained. The items retained for each factor along with the fit statistics for each model are shown in Table 2. Factor loadings for each item were high (above 0.5). All factor loadings were significant (*p* < 0.05). From our CFA, we confirmed that our instrument was measuring distinct and identifiable factors.

#### 3.1.2. Structural Equation Model (SEM)

**Figure 1:**
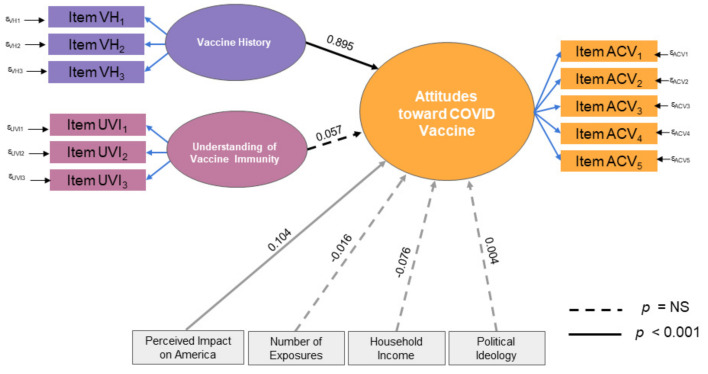
Design and results of the structural equation model. Vaccine history (VH) and Perceived Impact on America significantly predicted COVID-19 vaccine attitudes (ACV). Understanding of vaccine immunity (UVI) was not predictive. Of the covariates, the Perceived Impact on America was predictive, but not other demographic factors or personal relationships with infected individuals. The specific questions represented in the model are listed in Table 2.

Using SEM, we found that the two significant predictors of attitudes toward the COVID-19 vaccine are vaccine history and perceived impact of COVID-19 on America. In other words, respondents who routinely got vaccines were more likely to be receptive to receiving the COVID-19 vaccine. Additionally, the greater the perceived impact of COVID-19 on America, the more receptive the respondent was to receiving a potential COVID-19 vaccine. Interestingly, contrary to our predictions, an understanding of vaccines and immunity had no impact on the respondents’ attitudes; the number of people they knew with COVID-19 also appeared to be non-influential on their decisions. Household income and political ideology showed no relationship with attitudes toward the COVID-19 vaccine. The structural model showed a robust fit for the data as indicated by fit statistics and probability scores (RMSEA = 0.07, CFI = 0.93, TLI = 0.91, SRMR = 0.07, C^2^ = 1656.22, *p* < 0.001). The model with standardized estimates for relationships is shown in Figure 1.

### 3.2. Attitudes about COVID-19 and a Potential Vaccine

**Figure 2:**
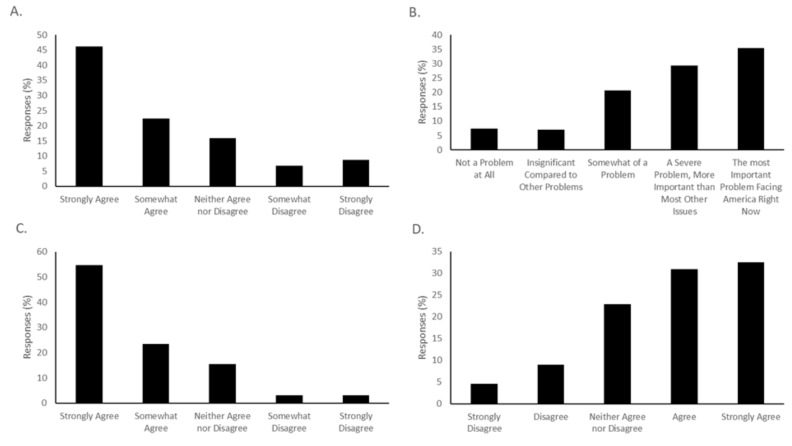
Vaccine acceptance, COVID-19 importance, and side effect concerns in the study population. (**A**) Intent to vaccinate. Survey participants were presented with the statement “I am likely to be vaccinated when a vaccine for COVID-19 becomes available” and asked to choose how they felt about the statement, ranging from “strongly agree” to “strongly disagree.” In total, 68.54% either chose “agree” or “strongly agree.” (**B**) Participants were asked to rate how much of a problem COVID-19 is in America. (**C**) Participants were provided the statement “A vaccine is important to end the COVID-19 pandemic,” and asked to rate their level of agreement. (**D**) Participants were given the statement “I am worried about side effects of a vaccine for myself” and asked to rate how much they agreed with the statement.

Most study subjects were agreeable towards vaccination for COVID-19, although only 46.11% of respondents “strongly agreed” with the statement “I am likely to be vaccinated for COVID-19 when a vaccine becomes available.” (Figure 2A). Another 22.46% chose “somewhat agree” when presented with that statement, for a total of 68.57% of respondents indicating that they were amenable to receiving a vaccine. 8.72% strongly disagreed that they would be vaccinated, and another 6.85% somewhat disagreed with vaccination. In total, 15.89% neither agreed nor disagreed. The respondents overall had a positive attitude towards the importance of a COVID-19 vaccine, with 54.83% selecting “strongly agree” and 23.36% selecting “somewhat agree” with the statement “A vaccine is important to stop the COVID-19 pandemic.” Only 3.12% selected “Strongly Disagree”, and 3.12% selected “Somewhat disagree” for this statement (Figure 2C).

Respondents mostly took the COVID-19 pandemic seriously. When asked to choose a response, 35.49% selected that the pandemic was “The most important problem facing America right now,” 29.32% selected “A severe problem, more important than most others,” and 20.68% chose “Somewhat of a problem.” Only 7.41% of respondents felt that the COVID-19 pandemic was “Not a problem at all,” while 7.10% chose “Insignificant compared to other problems.” (Figure 2B). Concern about the side effects of a vaccine was a serious issue. A majority (63.47%) of respondents either answered “Strongly Agree” or “Somewhat Agree” when given the statement “I am worried about the side effects of the vaccine for myself” (Figure 2D). A substantial number of respondents (although not a majority) worried that the side effects of a potential vaccine would be worse than the disease itself. When presented with the statement “The side effects of the vaccine are likely to be worse than COVID-19 itself,” 19.81% selected “strongly agree” and 19.50% selected “Somewhat agree.” In total, 14.86% selected “strongly disagree,” 16.72% selected “somewhat disagree” and 29.10% neither agreed nor disagreed.

### 3.3. Time Spent in Clinical Testing

**Figure 3:**
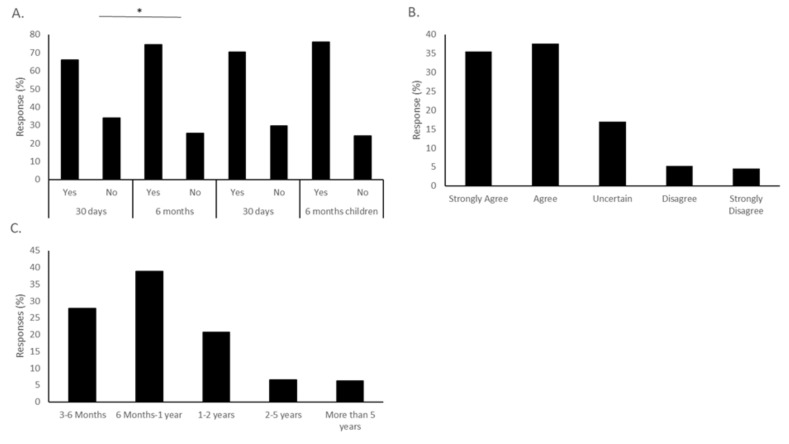
The time frame of vaccine testing influences the intent to vaccinate. (**A**) When asked if they would vaccinate themselves if a vaccine were available in the next 30 days, 66.05% of respondents answered affirmatively. When the time frame for vaccine availability was extended to 6 months, the number of people willing to be vaccinated significantly increased to 74.38% (* *p* = 0.02). The number of respondents willing to vaccinate their children also increased with increasing time to availability, but not significantly so. (**B**) Study participants were asked how much they agreed with the statement “I worry that the rushed pace of testing for a new COVID-19 vaccine will fail to detect potential side effects or dangers.” In total, 35.49% “strongly agreed,” and 37.65% “agreed” with this statement. (**C**) When asked “What is the minimum length of time a testing process would take that would make you feel comfortable with a COVID-19 vaccine?” the most common answer was 6 months to 1 year, at 38.89%; 3–6 months was second, at 27.78%, and 1–2 years was third, at 20.68%.

Respondents were asked about conditions that would affect their enthusiasm for a vaccine. When asked about the length of time a potential vaccine should be tested, 66.05% said they would be vaccinated if a vaccine was available in the next 30 days. The number of people willing to be vaccinated increased to 74.38% if the time span before a vaccine was available was extended to 6 months, a statistically significant increase (χ^2^ = 5.38, *p* = 0.02) (Figure 3A).

These same questions about timing were asked regarding the respondents’ willingness to vaccinate their children. Of the respondents with children, 70.45% were willing to vaccinate their children after a 30-day period, while 76.02% were willing to vaccinate their children if the vaccine was available in the next 6 months. Interestingly, this number is higher than those willing to vaccinate themselves within the same time frame of vaccine availability (Figure 3A).

The respondents who answered that they were unwilling to be vaccinated at each time frame were asked the reason why. For the 30-day time period, concerns about vaccine safety were the most commonly cited (45.45%), followed by “Other” (15.45%), and lack of trust in the source that encouraged them to receive the vaccine (13.54%). Of the respondents that selected “Other” and provided a reason, 10/16 indicated that they would need more testing before accepting the vaccine. Given a 6-month time frame, the reasons provided for not receiving the vaccine were not significantly different, although the number of refusals decreased.

To further understand the respondents’ attitudes to the connection between time to test the vaccine and vaccine safety, two other questions were asked. In response to the statement “I worry that the rushed pace of testing for a new COVID-19 vaccine will fail to detect potential side effects or dangers,” 35.49% strongly agreed, and 37.65% agreed, indicating that this was a concern for 73.14% of respondents (Figure 3B). In contrast, only 9.88% either disagreed or strongly disagreed with this statement. The second question asked the minimum length of time the vaccine would need to be tested for the respondents to be comfortable with the process. The most common answer was 6 months to a year, with 38.84% choosing this answer. The second most common selection was 3–6 months (27.78%), followed by 1–2 years (20.68%). Longer than two years was not often selected, with only 6.48% choosing 2–5 years, and 6.17% more than 5 years (Figure 3C).

When given a free-response option to identify their biggest fear about a potential COVID-19 vaccine, 51.85% of the meaningful responses expressed concerns about safety or side effects. Effectiveness was the second most expressed concern, at 10.65%. Not enough testing was third, with 10.18%. Finances were the primary concern for 2.77%. In contrast to those who worried about testing proceeding too rapidly, 2.31% were most concerned that the vaccine would not arrive in time, and 2.13% were afraid there would not be enough vaccine to go around or that there would be problems with distribution. Less than 1% were most worried about being tracked by microchips, the vaccine being made in China, or other people being unwilling to be vaccinated. Some people (10.65%) expressed that they had no concerns.

Study participants were also asked what would make them feel safest about a potential COVID-19 vaccine. The largest number of responses (31.06%) were about sufficient testing, 21.96% were about sufficient efficacy, and a known lack of side effects were mentioned 13.63% of the time. Nearly 7% of respondents would feel better if they thought the country was ready for a vaccine. Less than 1% ranked equity in distribution, the vaccine being made in the United States, having more information about the vaccine, or the vaccine being affordable as their item that would make them feel most secure. A large number (23.48%) said that they were unsure or that nothing would make them feel safe.

### 3.4. Vaccine Efficacy

**Figure 4:**
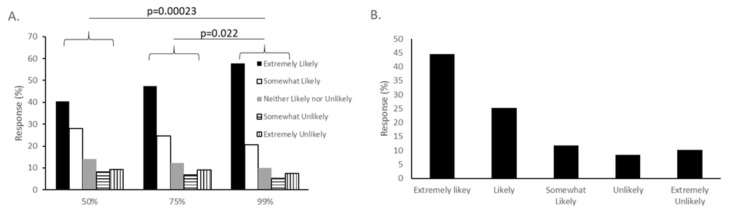
Vaccine efficacy contributes to intent to vaccinate. (**A**) Survey participants were given three scenarios, where the hypothetical vaccine was 50% effective, 75% effective, or 99% effective, and asked to rank how likely they were to be vaccinated under each scenario. The 99% effective vaccine was significantly better received than the other two, with 78.26 of respondents either somewhat or extremely likely to be vaccinated (*p* = 0.022 compared to 75%, *p* = 0.00023 compared to 50%). (**B**) Participants were asked about their attitudes towards a vaccine needing to be re-administered each year. There was no significant difference between intent to use a yearly vaccine and overall intent to be vaccinated.

Survey respondents were also concerned about vaccine efficacy. To evaluate this concern, we asked about three different levels of efficacy, and also about attitudes towards a vaccine that would need to be repeated yearly. Subjects were presented with three different hypothetical levels of efficacy: a vaccine that protected 50%, 75% or 99% of those immunized. When told the vaccine would protect 50% of vaccinated people, 40.58% of respondents said they would be extremely likely to vaccinate themselves, and 28.04% said they would be somewhat likely; 9.35% said they would be very unlikely and 8.10% said they would be somewhat unlikely to receive the vaccine. With a 75% efficacy rate, the numbers increased, with 47.35% selecting “extremely likely” and 24.61% falling in the “somewhat likely” group, although this difference between groups was not statistically significant (*p* = 0.171). When subjects were presented with a 99% effective rate, there was a statistically significant difference seen compared to the other groups. In this case, 56.70% chose “extremely likely” and 20.56% chose “somewhat likely.” Repeated measures ANOVA indicated a significant difference in variance between the three groups (*p* = 1.5 × 10^−10^). The 99% effective question showed a significant increase in likelihood to vaccinate compared to either the 50% (*p* = 0.00023) or the 75% (*p* = 0.022) questions. (Figure 4A).

Another aspect of vaccine efficacy is the length of immunity, as indicated by the need for repeated booster immunizations. When asked if they would be likely to be vaccinated if the vaccine required yearly boosters, 44.44% of respondents said it was extremely likely, and 25.31% said it was likely. Only 18.42% said that they would be unlikely or extremely unlikely to be vaccinated yearly (Figure 4B). There was no significant difference in attitudes towards a COVID-19 vaccine that needed to be administered yearly and the overall attitude towards a COVID-19 vaccine.

### 3.5. Location of Vaccine Development

**Figure 5:**
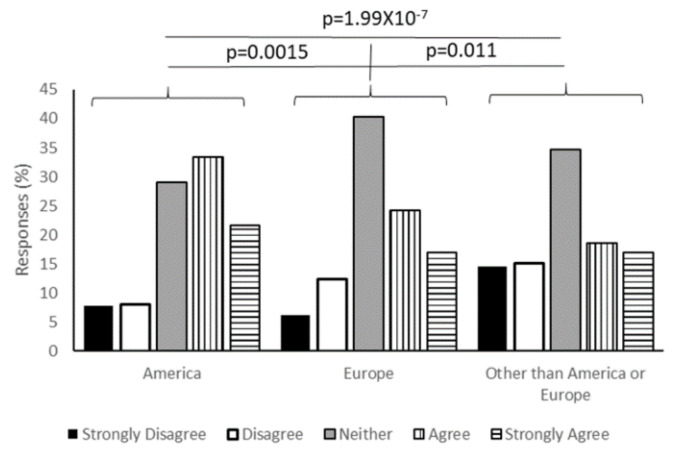
Location of vaccine development influences willingness to vaccinate. Survey respondents were asked to rate the statement “Knowing a COVID-19 vaccine was developed in America would make me feel more comfortable receiving it” from “Strongly Disagree” to “Strongly Agree.” There was a significantly higher level of comfort with an American-developed vaccine compared to a vaccine developed either in Europe (*p* = 0.0015) or “Other” locations (*p* = 1.99 × 10^−7^). European development was also favored over “Other” (*p* = 0.011).

Considering that Russia is promoting a vaccine, that there are several vaccines in development in China, and European, and that American institutions are all in late-stage vaccine development, it was important to understand the influence of where the vaccine is developed on acceptance of the vaccine in the United States. To this end, we asked for responses to three different statements: “Knowing a COVID-19 vaccine was developed in America would make me feel more comfortable receiving it.” “Knowing a COVID-19 vaccine was developed in Europe would make me feel more comfortable receiving it” and “Knowing a COVID-19 vaccine was developed somewhere other than America or Europe would make me feel more comfortable receiving it.” More recipients were comfortable with an American-made vaccine than any other location, with 21.67% of respondents selecting “Strongly agree” and 33.44% selecting “Agree.” For a European-developed vaccine, 17.03% strongly agreed and 24.15% agreed that it would make them comfortable. For other locations, the numbers were 17.03% strongly agree and 18.58% agree. Repeated-measures ANOVA indicated that there was significant variance between responses (*p* = 1.52 × 10^−12^). Confidence in an American-developed vaccine was significantly higher than a European vaccine (*p* = 0.0015) or one developed in another location (*p* = 1.99 × 10^−7^). There was also slightly more confidence in a potential European-developed vaccine than one developed in places other than Europe or America (*p* = 0.011) (Figure 5).

### 3.6. Types of Vaccine

Multiple different types of COVID-19 vaccines are currently in development, and the type of vaccine may influence the public’s attitudes towards vaccination. We asked the respondents about their level of comfort with attenuated, inactivated, subunit, RNA and vector-based vaccines. A brief explanation of each type was included with the question. The type of vaccine did not matter to the respondents, as we observed no significant difference in comfort.

### 3.7. Demographic Factors

In this study, no predictive value was found by the SEM model between COVID-19 vaccine attitudes and demographic factors. There was no predictive effect based on race, sex, age, income level or political affiliation. Political affiliation was evaluated using two questions: “What is your political affiliation” with the choices of Democrat, Republican or Independent, and “Please select the option that best describes your political ideology” with the possible choices Very conservative, Somewhat conservative, Neither conservative nor liberal, Somewhat liberal, and Very liberal. Neither the SEM model nor correlational analysis between political affiliation and intent to vaccinate showed a significant influence of political affiliation, nor did ANOVA analysis of intent to vaccinate values when separated into groups by political affiliation.

**Figure 6:**
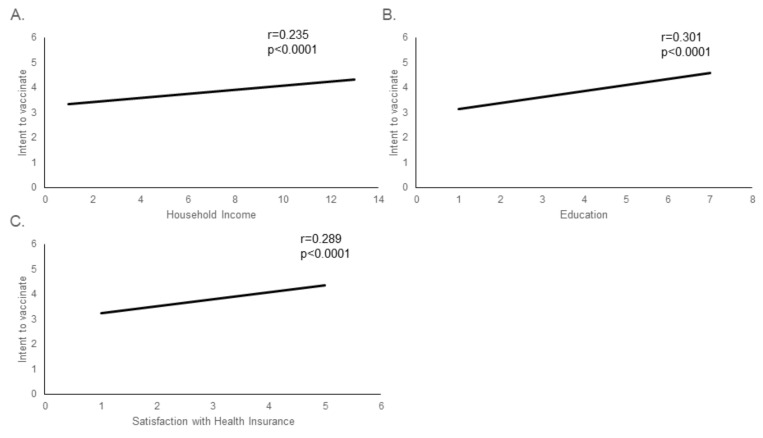
Economic indicators correlate with intent to vaccinate. Three survey questions (**A**: “Which category best describes your yearly household income before taxes? Include all income received from employment, social security, support from children or other family, welfare, Aid to Families with Dependent Children (AFDC), bank interest, retirement accounts, rental property, investments, etc.”, **B**: “Level of Education”, and **C**: “How would you rate your health insurance?”) were compared to the responses to the statement “I am likely to be vaccinated for COVID-19 when a vaccine becomes available” (Intent to vaccinate). All three economic measures were strongly positively correlated with intent to vaccinate (*p* < 0.0001 for each). Due to the large number of responses, only the trendlines are shown for each correlation.

However, when examined individually, income level, education and satisfaction with health insurance all significantly correlated with intent to vaccinate. (Figure 6).

### 3.8. Personal Relationships with COVID-19 Patients

The majority of respondents (61.08%) knew someone who had tested positive for SARS-CoV-2. In total, 21.52% of respondents knew more than one person who tested positive. The most common relationships were friends and friends of friends. We also measured the seriousness of the resulting disease, from no symptoms to severe hospitalization to death, for each relationship. Moderate symptoms without hospitalization were the most common outcome. Despite the high incidence of COVID-19 among associates of the respondents, there was no statistical association of COVID-19 vaccine attitudes with either the closeness of the relationship or the seriousness of the disease. Those who knew multiple people with the disease were no more likely to intend to vaccinate than those who did not have a relationship with someone with COVID-19.

### 3.9. COVID-19 Knowledge

To test the respondents’ knowledge about COVID-19 and SARS-CoV-2, we asked for responses to eight true/false questions, then added up the correct answers for a total knowledge score. The questions were derived from the WHO coronavirus information page. The knowledge score did not significantly correlate with intent to vaccinate, fear of side effects or the response to the question “How closely do you follow news regarding COVID-19”. The primary source of news about COVID-19 did not correlate with the knowledge score.

## 4. Discussion

Most people in the United States rank COVID-19 as a severe problem, and most of them view a vaccine against COVID-19 as necessary and something they are willing to receive. However, even with a majority of people accepting of the vaccine, the number of enthusiastic or highly enthusiastic people still falls short of ideal, and possibly short of the numbers necessary to stop the pandemic. Several factors contribute to concerns about the vaccine, and a vaccine development and promotion strategy that addresses these concerns would be useful in increasing participation in a vaccination campaign.

Vaccine history was the most important predictor of the intent to receive a COVID-19 vaccine, a factor which reflects confidence in vaccines in general. This finding is reinforced by the concern about side effects seen throughout the responses. Therefore, any effective measures promoting information about the safety of vaccines or improving vaccine acceptance should also aid in COVID-19 vaccine acceptance. Efforts to improve the transparency and thoroughness of testing will likely improve vaccine usage. These results are particularly relevant given the current push towards the early release of a vaccine, which, if it appears politically motivated or results in significant side effects, may have the effect of decreasing confidence in vaccines in general [20].

The other predictor of COVID-19 vaccine acceptance was the perception that COVID-19 is a serious problem for the United States. This finding suggests that efforts to emphasize the consequences of the pandemic on the overall well-being of the United States, including the economic, social and public costs of the disease, will likely be effective in encouraging vaccination. Interestingly, political affiliation was not predictive of intent to vaccinate, suggesting that the decision to be vaccinated is not political, and therefore members of all political ideologies may be receptive to messages aimed at increasing COVID-19 vaccination. As federal elections in the United States become nearer and more and more is politicized, this could change.

Several of our original hypotheses were not supported by the results of the study. Our prior work showed that personal stories of people who had suffered vaccine-preventable diseases were effective in improving vaccine attitudes in vaccine-hesitant individuals. However, in this study, relationships with people who had tested positive did not predict vaccine attitudes. It may be the case that the overwhelming nature of the pandemic dilutes the effects of personal stories, since nearly everyone is affected to some degree. It may also be the case that, since the impact of COVID-19 on the United States was a predictor of intent to vaccinate, people are viewing this disease as a societal issue more than a personal one.

We had also predicted that specific knowledge of the disease, or of a specific type of vaccine, would affect intent to vaccinate, which was not supported by the survey results. It seems that specific details are not extremely important in the case of attitudes towards a vaccine for COVID-19. Instead, general pro-vaccination principles, such as sufficient testing, safety, efficacy and being “made in America” should be emphasized. Emphasis should also be placed on the societal good of vaccination, and the harms of the uncontrolled disease should be emphasized.

Given that the harms of the pandemic are falling disproportionately on the low-income sector, special efforts should be made to ensure that this proportion of the population has access to vaccination. Our data also show that lower-income people may be less likely to intend to be vaccinated, so messages addressing their specific concerns would likely be effective both in serving the most affected population and in increasing overall vaccine usage.

The major limitation of the study is the timing. During the two-week period between when the survey was released and the manuscript was submitted, a push for accelerated vaccine deployment was announced by the CDC, and a major vaccine trial was paused due to safety concerns. The survey, therefore, does not reflect these two fairly significant occurrences. However, the findings of the survey should still apply, and will likely be heightened by these events. The number of respondents is also less than in some surveys of this type, although our model fit statistics were robust.

A repetition of similar surveys would be useful to understand the changing attitudes towards a COVID-19 vaccine as both a vaccine and political elections become closer.

## 5. Conclusions

Through this study, we found two factors that significantly predicted the respondents’ attitudes towards a potential COVID-19 vaccine. We also found significant effects of timing, efficacy and location on willingness to be vaccinated. Efforts to address these factors, including design of potential vaccines, testing of these vaccines, and directed public outreach efforts, will likely improve vaccine usage, contributing to control of the COVID-19 pandemic.

## Figures and Tables

**Table 1:** Demographics of the study population.

	Number	Percentage
AGE		
Less than 18	7	2.16
18–25	40	12.35
26–35	59	18.21
36–45	102	31.48
46–55	11	3.4
Over 55	105	32.41
RACE/ETHNICITY		
American Indian or Alaska Native	4	1.23
Asian	17	5.25
Black or African American	39	12.04
Hispanic or Latino	54	16.67
Native Hawaiian or Pacific islander	1	0.31
White	205	63.27
Other	0	0
Prefer not to Answer	4	1.23
SEX		
Female	160	49.38
Male	159	49.07
Non-binary/Third Gender	2	0.62
Prefer to self-describe	1	0.31
Prefer not to answer	2	0.62

**Table 2:** Fit statistics for each latent variable and full measurement model.

Latent Variable and Associated Items (Factor Loading Indicated ^a^)	RMSEA	CFI	TLI	SRMR	Chi-Square Test		
					C^2^	DF	*p*-Value
Vaccine History	0.00	1.00	1.00	0.00	84.31	3	<0.001
*I am current on the vaccinations recommended by my primary care physician. ^b^ (0.53)*							
*How important is it for you to get the flu vaccine every year? ^b^ (0.69)*							
*My children are current on which recommended vaccines (or, if I don’t have children, I would keep my children current on which recommended vaccines)? ^b^ (0.65)*							
Understanding of Vaccine Immunity	0.00	1.00	1.00	0.00	180.86	3	<0.001
*I would rather build immunity by exposure to an infected individual than receive the vaccine. ^c^ (0.83)*							
*Not everyone who is eligible for the vaccine needs to receive it because herd immunity is sufficient to protect everyone. ^c^ (0.80)*							
*The side effects of the vaccine are likely to be worse than COVID-19 itself. ^c^ (0.76)*							
Attitudes toward COVID-19 Vaccine	0.11	0.97	0.93	0.04	547.99	10	<0.001
*If a COVID-19 vaccine was made publicly available, but it would need to be administered yearly (similar to the flu shot), how likely would you be to be vaccinated? ^b^ (0.90)*							
*If a vaccine for COVID-19 was made available and you were told it would protect half of the people who received it, how likely would you be to be vaccinated? ^b^ (0.90)*							
*Other people being vaccinated against COVID-19 will be helpful in controlling the pandemic. ^c^ (0.70)*							
*I am likely to be vaccinated for COVID-19 when a vaccine becomes available. ^c^ (0.87)*							
*A vaccine is important to end the COVID-19 pandemic. ^c^ (0.70)*							
Complete Measurement Model	0.07	0.95	0.94	0.05	1370.56	55	<0.001

^a^ All factor loadings are standardized; ^b^ These items were on a 5-point Likert scale indicating a low value to a high value (for specific statements, see full survey in the Supplementary Materials); ^c^ These items were on a 5-point Likert scale indicating level of agreement (for specific statements, see full survey in the Supplementary Materials).

## Supplementary Materials

The following are available online at https://www.mdpi.com/2076-393X/8/4/582/s1, Supplementary Materials: Complete survey.
