# Supplementary material for: Influences on Attitudes Regarding Potential COVID-19 Vaccination in the United States
Source: Vaccines (Basel). 2020 Oct 3;8(4):582. doi: 10.3390/vaccines8040582 (PMC7711655; doi:10.3390/vaccines8040582)
Supplement: Supplementary file 1 [file vaccines-08-00582-s001.pdf]

Covid-19 survey

Survey Flow

EmbeddedData
opp = Qual5045-0805-COVIDVaccine
Q_TotalDurationValue will be set from Panel or URL.
qpmid = 4519
ridValue will be set from Panel or URL.
RISNValue will be set from Panel or URL.
LSValue will be set from Panel or URL.

Branch: New Branch
If
If Quota Overall (300) Has Been Met

EmbeddedData
gc = 3
term = quota_full

EndSurvey: Advanced

Standard: Part 2A (12 Questions)

Branch: New Branch
If
If Quota Overall (300) Has Been Met

EmbeddedData
gc = 3
term = quota_full

EndSurvey: Advanced

Standard: Part 2B (4 Questions)
Standard: Part 2C (2 Questions)
Standard: Part 2C (second half) (9 Questions)
Standard: Part 2D (2 Questions)
Standard: Part 2E (15 Questions)
Standard: Part 2F (7 Questions)
Standard: Part 2G (8 Questions)
Block: part 1: Demographic information (5 Questions)
Standard: part 1: Socioeconomic status (7 Questions)

Branch: New Branch
If
If Q_TotalDuration Is Less Than 162

EmbeddedData
gc = 4
term = Speeder

EndSurvey: Advanced

EmbeddedData
gc = 1
LS = $e{(${e://Field/RISN}%3402)*3}

EndSurvey: Advanced

Page Break

Start of Block: Part 2A

Q93 Thank you for taking this survey. This survey is about COVID-19, and ways of dealing with the current pandemic.

Q1.2 Age

- O Less than 18 (1)
- O 18-25 (2)
- O 26-35 (3)
- O 36-45 (4)
- O 46-55 (5)
- O Over 55 (6)

Q1.4 Sex

- O Male (1)
- O Female (2)
- O Non-binary/third gender (3)
- O Prefer to self-describe (4) ____
- O Prefer not to answer (5)

Q1.3 Race

- American Indian or Alaska Native (1)
- Asian (2)
- Black or African American (3)
- Hispanic or Latino (4)
- Native Hawaiian or Pacific Islander (5)
- White (6)
- Other (7) ____
- Prefer not to answer (8)

Page Break

Q3.2 Please select ALL of the following statements that apply to your experience with COVID-19:

- ☐ I have tested positive for a COVID-19 infection (1)
- ☐ An immediate family member has tested positive for COVID-19 infection (2)
- ☐ An extended family member has tested positive for COVID-19 infection (3)
- ☐ A friend who does not live with me has tested positive for COVID-19 infection (4)
- ☐ A roommate or friend who lives with me has tested positive for COVID-19 infection (5)
- ☐ A coworker has tested positive for COVID-19 infection (6)
- ☐ A distant friend or a friend of a friend has tested positive for COVID-19 infection (7)
- ☐ I do not know anyone who has tested positive for COVID-19 infection (8)

Display This Question:

If Q3.2 = I have tested positive for a COVID-19 infection

Q3.3 How severe was *your COVID-19 infection*?

- O No symptoms/mild symptoms (1)
- O Moderate symptoms but health care providers were not contacted (2)
- O Moderate symptoms and health care providers were contacted (3)
- O Severe symptoms/hospitalization (4)

Display This Question:

If Q3.2 = An immediate family member has tested positive for COVID-19 infection

Q3.4 How severe were the symptoms of the COVID-19 infection of *your immediate family member?*

- No symptoms/mild symptoms (1)
- Moderate symptoms but health care providers were not contacted (2)
- Moderate symptoms and health care providers were contacted (3)
- Severe symptoms/hospitalization (4)
- Death (5)

Display This Question:

If Q3.2 = An extended family member has tested positive for COVID-19 infection

Q3.5 How severe were the symptoms of the COVID-19 infection of *your extended family member?*

- No symptoms/mild symptoms (1)
- Moderate symptoms but health care providers were not contacted (2)
- Moderate symptoms and health care providers were contacted (3)
- Severe symptoms/hospitalization (4)
- Death (5)

Display This Question:

If Q3.2 = A friend who does not live with me has tested positive for COVID-19 infection

Q3.6 How severe were the symptoms of the COVID-19 infection of *your friend who does not live with you?*

O No symptoms/mild symptoms (1)

O Moderate symptoms but health care providers were not contacted (2)

O Moderate symptoms and health care providers were contacted (3)

O Severe symptoms/hospitalization (4)

O Death (5)

Display This Question:

If Q3.2 = A roommate or friend who lives with me has tested positive for COVID-19 infection

Q3.7 How severe were the symptoms of the COVID-19 infection of *the roommate or friend you live with?*

O No symptoms/mild symptoms (1)

O Moderate symptoms but health care providers were not contacted (2)

O Moderate symptoms and health care providers were contacted (3)

O Severe symptoms/hospitalization (4)

O Death (5)

Display This Question:

If Q3.2 = A coworker has tested positive for COVID-19 infection

Q3.8 How severe were the symptoms of the COVID-19 infection of *your coworker?*

- O No symptoms/mild symptoms (1)
- O Moderate symptoms but health care providers were not contacted (2)
- O Moderate symptoms and health care providers were contacted (3)
- O Severe symptoms/hospitalization (4)
- O Death (5)

Display This Question:

If Q3.2 = A distant friend or a friend of a friend has tested positive for COVID-19 infection

Q3.9 How severe were the symptoms of the COVID-19 infection of *your distant friend or friend of a friend?*

- O No symptoms/mild symptoms (1)
- O Moderate symptoms but health care providers were not contacted (2)
- O Moderate symptoms and health care providers were contacted (3)
- O Severe symptoms/hospitalization (4)
- O Death (5)

End of Block: Part 2A

Start of Block: Part 2B

Q4.1 How has your experience with the COVID-19 global pandemic affected your opinion on vaccinations in general (not including a potential COVID-19 vaccine)?

- I am much more likely to vaccinate myself/my children (1)
- I am more likely to vaccinate myself/my children (2)
- My opinion on vaccinations has not changed (3)
- I am less likely to vaccinate myself/my children (4)
- I am much less likely to vaccinate myself/my children (5)

Q4.2 How has the COVID-19 pandemic affected your employment/income?

- Severely (1)
- Moderately (2)
- Mildly (3)
- Not at all (4)
- Improved (5)

Q4.3 How has the COVID-19 pandemic affected your mental health?

- Much better (1)
- Somewhat better (2)
- About the same (3)
- Somewhat worse (4)
- Much worse (5)

Q4.4 How has the COVID-19 pandemic affected your ability to carry out your normal activities?

- O Dramatic restrictions (1)
- O Moderate restrictions (2)
- O No restrictions (3)
- O Moderately easier (4)
- O Much easier (5)

End of Block: Part 2B

Start of Block: Part 2C

Q5.2 How closely do you follow news regarding COVID-19?

- O Very closely (1)
- O Somewhat closely (2)
- O An average amount (3)
- O Not very closely (4)
- O Not at all (5)

Q5.3 What is your primary source of information regarding COVID-19?

- O Your primary doctor (1)
- O CDC (Center for Disease Control and Prevention), WHO (World Health Organization) or local Board of Health (2)
- O Local news (3)
- O Friends or Social Media (4)
- O Celebrities/public figures (6)
- O Religious leaders (7)
- O Political leaders (9)
- O Other (Please specify) (8) ____

End of Block: Part 2C

Start of Block: Part 2C (second half)

Carry Forward Selected Choices from "Q6.1"

Q6.1 Please indicate whether the following statements are true or false:

Q6.2 Vaccines against pneumonia can protect against COVID-19

- O True (1)
- O False (2)

Q6.3 Certain antibiotics can prevent and/or treat COVID-19.

O True (1)

O False (2)

Q6.4 On average it takes 5–6 days from when someone is infected with COVID-19 for symptoms to show, however it can take up to 14 days.

O True (1)

O False (2)

Q6.5 Regularly rinsing your nose with saline can help prevent infection with COVID-19.

O True (1)

O False (2)

Q6.6 Once you contract COVID-19, the virus can never be eliminated from your body.

O True (1)

O False (2)

Q6.7 Symptoms of COVID-19 can include sore throat, diarrhea, and conjunctivitis (eye infection).

O True (1)

O False (2)

Q6.8 COVID-19 can be killed by hand dryers.

- O True (1)
- O False (2)

Q6.9 Most people who contract COVID-19 will recover from it.

- O True (1)
- O False (2)

End of Block: Part 2C (second half)

Start of Block: Part 2D

Q7.1 I am current on the vaccinations recommended by my primary care physician.

- O I am current on all (1)
- O I am current on most (2)
- O I am current on some (3)
- O I am not current on any (4)
- O I am uncertain (5)

Q7.3 How important is it for you to get the flu vaccine every year?

- O Very important (1)
- O Important (2)
- O Somewhat important (3)
- O Not very important (4)
- O Not at all important (5)

End of Block: Part 2D

Start of Block: Part 2E

Q8.1 Please indicate how you would respond in the following scenarios if a vaccine were developed to prevent contracting COVID-19.

Q8.2 If a COVID-19 vaccine was made publicly available in the next 30 days...

- O I would vaccinate myself (1)
- O ⊗ I would not vaccinate myself (2)

Display This Question:

If Q8.2 = I would not vaccinate myself

Q8.3 Which of these statements most closely resembles your reason for choosing to not vaccinate yourself:

- O I do not believe the vaccine is safe (1)
- O I do not believe the vaccine is effective (2)
- O I do not trust the source that encouraged me to get the vaccine (3)
- O I do not believe in any vaccines, and my reason is not any different for a new COVID-19 vaccine (4)
- O A source that I trust encouraged me to NOT get the vaccine (5)
- O I am indifferent to receiving the vaccine, but will probably end up not receiving it (6)
- O Other (7) ____

Q8.4 If a COVID-19 vaccine was made publicly available in the next 30 days...

- O I do have children and I would vaccinate them (1)
- O I do have children and I would not vaccinate them (2)
- O I do not have children (3)

Display This Question:

If Q8.4 = I do have children and I would not vaccinate them

Q8.5 Which of these statements most closely resembles your reason for choosing to not vaccinate your children:

- O I do not believe the vaccine is safe for children (1)
- O I do not believe the vaccine is effective (2)
- O I do not trust the source that encouraged me to give my child the vaccine (3)
- O I do not believe in any vaccines, and my reason is not any different for a new COVID-19 vaccine (4)
- O A source that I trust encouraged me to NOT give my child the vaccine (5)
- O I am indifferent to having my child receive the vaccine, but probably will not end up having my child receive it (6)
- O Other (7) ____

Q8.6 If a COVID-19 vaccine was made publicly available after the next 6 months...

- O I would vaccinate myself (1)
- O ⨂ I would not vaccinate myself (2)

Display This Question:

If Q8.6 = I would not vaccinate myself

Q8.7 Which of these statements most closely resembles your reason for choosing to not vaccinate yourself:

- I do not believe the vaccine is safe (1)
- I do not believe the vaccine is effective (2)
- I do not trust the source that encouraged me to get the vaccine (3)
- I do not believe in any vaccines, and my reason is not any different for a new COVID-19 vaccine (4)
- A source that I trust encouraged me to NOT get the vaccine (5)
- I am indifferent to receiving the vaccine, but will probably end up not receiving it (6)
- Other (7) ____

Q8.8 If a COVID-19 vaccine was made publicly available after the next 6 months...

- I do have children and I would vaccinate them (1)
- I do have children and I would not vaccinate them (2)
- I do not have children (3)

Display This Question:

If Q8.8 = I do have children and I would not vaccinate them

Q8.9 Which of these statements most closely resembles your reason for choosing to not vaccinate your children:

- I do not believe the vaccine is safe for children (1)
- I do not believe the vaccine is effective (2)
- I do not trust the source that encouraged me to give my child the vaccine (3)
- I do not believe in any vaccines, and my reason is not any different for a new COVID-19 vaccine (4)
- A source that I trust encouraged me to NOT give my child the vaccine (5)
- I am indifferent to having my child receive the vaccine, but probably will not end up having my child receive it (6)
- Other (7) ____

Q8.10 If a COVID-19 vaccine was made publicly available, but it would need to be administered yearly (similar to the flu shot), how likely would you be to be vaccinated?

- Extremely likely (almost every year) (1)
- Very likely (2)
- Somewhat likely (3)
- Not very likely (4)
- Not at all likely (almost never) (5)

Display This Question:

If Q8.10 = Not very likely

And Q8.10 = Not at all likely (almost never)

Q8.11 Which of the following most closely describes why you would be unlikely to receive a yearly vaccination for yourself?

- O Limited time (1)
- O Limited money or insurance (2)
- O Limited access to health care (3)
- O Concern with the vaccine itself (4)
- O Other (5) ____

Q8.12 If a vaccine for COVID-19 was made available and you were told it would protect half of the people who received it, how likely would you be to be vaccinated?

- O Extremely likely (1)
- O Somewhat likely (2)
- O Neither likely nor unlikely (3)
- O Somewhat unlikely (4)
- O Extremely unlikely (5)

---

q8.13 If a vaccine for COVID-19 was made available and you were told it would protect 3/4 of those who received it, how likely would you be to be vaccinated?

- O Extremely likely (1)
- O Somewhat likely (2)
- O Neither likely nor unlikely (3)
- O Somewhat unlikely (4)
- O Extremely unlikely (5)

Q90 If a vaccine for COVID-19 was made available and you were told it would protect 99% of those who received it, how likely would you be to be vaccinated?

- O Extremely likely (1)
- O Somewhat likely (2)
- O Neither likely nor unlikely (3)
- O Somewhat unlikely (4)
- O Extremely unlikely (5)

Q91
Other people being vaccinated against COVID-19 will be helpful in controlling the pandemic

- O Strongly agree (1)
- O Somewhat agree (2)
- O Neither agree nor disagree (3)
- O Somewhat disagree (4)
- O Strongly disagree (5)

End of Block: Part 2E

Start of Block: Part 2F

Q9.1 Vaccines are important for the prevention of serious diseases.

- O Strongly agree (1)
- O Agree (2)
- O Uncertain (3)
- O Disagree (4)
- O Strongly disagree (5)

Q9.2 The administration of more than one vaccine at the same time can be unsafe for my child's health.

- O Strongly agree (1)
- O Agree (2)
- O Uncertain (3)
- O Disagree (4)
- O Strongly disagree (5)

Q9.3 The side effects of most vaccines outweigh the benefits

- O Strongly agree (1)
- O Agree (2)
- O Uncertain (3)
- O Disagree (4)
- O Strongly disagree (5)

Q9.4 I worry that the rushed pace of testing for a new COVID-19 vaccine will fail to detect potential side effects or dangers.

- Strongly agree (1)
- Agree (2)
- Uncertain (3)
- Disagree (4)
- Strongly disagree (5)

Q9.6 My children are current on which recommended vaccines (or, if I don't have children, I would keep my children current on which recommended vaccines)?

- All recommended vaccines (1)
- Most recommended vaccines (2)
- Some recommended vaccines (3)
- Uncertain (4)
- My children are not/would not be vaccinated (5)

Q9.7 I am likely to be vaccinated for COVID-19 when a vaccine becomes available

- Strongly agree (1)
- Somewhat agree (2)
- Neither agree nor disagree (3)
- Somewhat disagree (4)
- Strongly disagree (5)

Q9.8 A vaccine is important to end the COVID-19 pandemic

- Strongly agree (1)
- Somewhat agree (2)
- Neither agree nor disagree (3)
- Somewhat disagree (4)
- Strongly disagree (5)

End of Block: Part 2F

Start of Block: Part 2G

Q10.1 What is the minimum length of time a testing process would take that would make you feel comfortable with a COVID-19 vaccine?

- 3-6 months (1)
- 6 months to a year (2)
- 1-2 years (3)
- Between 2 and 5 years (4)
- More than 5 years (5)

Q10.2 There are many types of vaccines. Read each type of vaccine below and rate it from 1-5 on how you feel about it as a logical, safe, and effective way to achieve immunity against COVID-19, 1 being not at all comfortable and 5 being totally comfortable. (Note, this is not asking about your comfort with vaccines in general, but rather your opinion about certain types of vaccines)

	1 (Not at All Comfortable) (1)	2 (Slightly Uncomfortable) (2)	3 (Neither comfortable nor Uncomfortable) (3)	4 (Slightly Comfortable) (4)	5 (Very Comfortable) (5)
Live vaccine (attenuated): This type of vaccine is made by weakening the virus so that it causes a very small infection; enough to activate your immune system but not enough to make you sick. (1)	o	o	o	o	o
Killed (Inactivated) vaccine: This type of vaccine is made by treating the virus with chemicals that make it non-functional. It does not infect you, but it may require more injections than a live vaccine (2)	o	o	o	o	o

Subunit vaccine: this type of vaccine uses pieces of the virus, usually made in yeast cells, to stimulate your immune system. There is no infection but it may take more than one immunization to become fully immune (3)	o	o	o	o	o
RNA vaccine: This type of vaccine uses RNA molecules to tell your cells to make a small piece of the virus. Your immune system is then activated against this piece of virus. This is newer technology, but will likely make a strong immune response (4)	o	o	o	o	o
Carrier vaccine: This type of vaccine uses a different	o	o	o	o	o

virus, that does not cause disease in humans, to carry a small piece of the COVID virus. It will cause a very small infection; enough to activate the immune system but not enough to make you sick (5)

Q10.3 Please rank from 1-5 how much you agree with the following statements, where 1 is Strongly Disagree and 5 is Strongly Agree

	1 (Strongly Disagree) (1)	2 (Disagree) (2)	3 (Neither agree nor Disagree) (3)	4 (Agree) (4)	5 (Strongly Agree) (5)
Receiving a COVID-19 vaccine at the same time as regularly scheduled vaccines would make me more likely to accept it. (1)	o	o	o	o	o
I am worried that the vaccine itself will give me COVID-19. (2)	o	o	o	o	o
I would rather build immunity by exposure to an infected individual than receive the vaccine. (3)	o	o	o	o	o
I would be more likely to get the vaccine if it was required to travel internationally. (4)	o	o	o	o	o
Not everyone who is eligible for the vaccine needs to receive it because herd immunity is sufficient to protect everyone. (5)	o	o	o	o	o

I am worried about the cost of a COVID-19 vaccine. (6)	o	o	o	o	o
I am worried about side effects of the vaccine for myself. (7)	o	o	o	o	o
I am worried about side effects of the vaccine for children. (8)	o	o	o	o	o
The side effects of the vaccine are likely to be worse than COVID-19 itself (9)	o	o	o	o	o
Knowing a COVID-19 vaccine was developed in America would make me feel more comfortable receiving it (10)	o	o	o	o	o
Knowing a COVID-19 vaccine was developed in Europe would make me feel more comfortable receiving it (11)	o	o	o	o	o
Knowing a COVID vaccine was developed	o	o	o	o	o

somewhere other than America or Europe would make me feel more comfortable receiving it. (12)

Q10.4 Please answer the following questions in your own words: The biggest fear I have about a COVID-19 vaccine is...

____

Q10.5 Please answer the following question in your own words: What would make you the most comfortable with the idea of receiving a vaccine for COVID-19?

____

Q10.6 I trust the following measures to protect myself and others from COVID-19 (select all that apply):

▢ Wearing a mask while in public (1)

▢ Other people wearing masks (2)

▢ Social Distancing (3)

▢ Public sanitation measures like frequent handwashing and disinfecting surfaces (4)

▢ None of the above (5)

Q10.7 I currently take the following measures to protect myself and others from COVID-19 (select all that apply):

- ▢ Wearing a mask while in public (1)
- ▢ Social distancing (2)
- ▢ Public sanitation measures like frequent handwashing and disinfecting surfaces (3)
- ▢ None of the above (4)

Q10.8 How much of a problem is COVID-19 in America?

- O Not a problem at all (1)
- O Insignificant compared to other problems (2)
- O Somewhat of a problem (3)
- O A severe problem, more important than most other issues (4)
- O The most important problem facing America right now (5)

End of Block: Part 2G

Start of Block: part 1: Demographic information

Q1.1 These questions are helpful to the researchers in comparing similar groups of people and to help us understand the results of the research better. Although these may be personal questions, your name and specific information will not be associated with the survey. Please select the best answer for each question.

Q1.5 Marital Staus

- O Single (1)
- O Married (2)
- O Divorced (3)
- O Widow/widower (4)

Q1.6 Number of children

- O 0 (1)
- O 1 (2)
- O 2 (3)
- O More than 2 (4)

Q1.7 Level of education

- O Have not finished high school (1)
- O Finished high school (2)
- O Some college (3)
- O Associates degree (4)
- O Bachelors degree (5)
- O Masters degree (6)
- O Doctoral degree (e.g., MD,DDS,PhD) (7)

Q1.8 Political affiliation

- Democrat (1)
- Republican (2)
- Independent (3)

End of Block: part 1: Demographic information

Start of Block: part 1: Socioeconomic status

Q2.2 How many bedrooms (including guest bedrooms, bedrooms used as offices, etc.) are in the house or apartment which is your primary residence?

- 1 (1)
- 2 (2)
- 3 (3)
- 4 (4)
- More than 4 (5)

Q2.3 What is your current employment status? Check ALL that apply.

- ☐ Working full time for pay (please specifiy number of hours per week) (1) ____
- ☐ Working part time for pay (please specify number of hours per week) (2) ____
- ☐ Not currently employed, looking for work (3)
- ☐ Retired (4)
- ☐ Homemaker (5)
- ☐ Disabled (not working because of a permanent or temporary disability) (6)
- ☐ Other (please specify) (7) ____

Q2.4 Which category best describes your yearly household income before taxes? Include all income received from employment, social security, support from children or other family, welfare, Aid to Families with Dependent Children (AFDC), bank interest, retirement accounts, rental property, investments, etc.

- Less than $5,000 (1)
- $5,000 - $9,999 (2)
- $10,000 - $14,999 (3)
- $15,000 - $19,999 (4)
- $20,000 - $29,999 (5)
- $30,000 - $39,999 (6)
- $40,000 - $49,999 (7)
- $50,000 - $59,999 (8)
- $60-000 - $74,999 (9)
- $75,000 - $99,999 (10)
- $100,000 - $124,999 (11)
- $125,000 - $149,999 (12)
- $150,000 or more (13)
- Prefer not to answer (14)

Q2.5 During the last year, did you take an out-of-town vacation?

- Yes (1)
- No (2)

Q2.6 Religious affiliation

- I am not affiliated with any faith tradition (1)
- Baptist (2)
- Catholic (3)
- Christian (non-denominational) (4)
- Church of Christ/Disciples of Christ (5)
- Congregational (6)
- Episcopalian/Anglican (7)
- Jehovah's Witness (8)
- Jewish (9)
- LDS (Mormon) (10)
- Lutheran (11)
- Methodist/Wesleyan (12)
- Orthodox (Eastern) (13)
- Pentecostal/Charismatic (14)
- Protestant (Other) (15)
- Reformed/Presbyterian (16)
- Seventh-day Adventist (17)
- Agnostic (18)
- Atheist (19)
- Other, please specify (20) ____________________

Q2.7 Please select the option that best describes your political ideology

- Very conservative (1)
- Somewhat conservative (2)
- Neither conservative nor liberal (3)
- Somewhat liberal (4)
- Very liberal (5)

Q92 How would you rate your health insurance?

- Excellent (1)
- Good (2)
- Average (3)
- Poor (4)
- Terrible (5)
- I do not have health insurance (6)

End of Block: part 1: Socioeconomic status
